# Supplementary material for: The volatility of beef and lamb prices in Türkiye: The role of COVID–19, livestock imports, and energy prices
Source: PLoS One. 2023 Mar 13;18(3):e0282611. doi: 10.1371/journal.pone.0282611 (PMC10010543; doi:10.1371/journal.pone.0282611)
Supplement: Attachment — Submitted filename: Feedback.docx [file pone.0282611.s004.docx]

**The volatility of beef and lamb prices in Turkey: The role of COVID-19, livestock imports, and energy prices**

Summary:

The paper addresses the impact of three variables (Covid-19, livestock import, and Energy prices) on beef and lamb prices in Turkey. Using data covering the period of April 2006 through February 2022, the study employs a VAR(1) asymmetric BEKK GARCH model for empirical analysis. The study finds different effects of different variables and offers some policy recommendations.

The paper addresses an important issue with an appropriate method. The results are interesting with sound discussion in the context of previous literature. The policy recommendations are relevant. The paper is well-structured, well-written, and well-organized.

Major issue

1. Some part of the method section is not self-explanatory and lacks some relevant information, which makes it hard for the reader to follow along. The reader is left alone in that regard. For example, it is not clear how the Covid period was singled out from the data period of 2006 and 2022.How was the COVID-19-induced uncertainties incorporated into the model? Was a dummy variable used(like the import variable, which is mentioned in this section)? If so, the method section does not indicate it. I could understand it after seeing the results in table 3. This should be clarified in the method section.

Minor

1. Line 39, “food safety” should be replaced by “food security”
2. Line 228, What do E and I denote for?
3. The official name of the country changed from “Turkey” to “Turkiye”. The new name could be used.
